# Domain Wall Evolution in Hf_0.5_Zr_0.5_O_2_ Ferroelectrics under Field-Cycling Behavior

**DOI:** 10.34133/research.0093

**Published:** 2023-03-28

**Authors:** Sirui Zhang, Qinghua Zhang, Fanqi Meng, Ting Lin, Binjian Zeng, Lin Gu, Min Liao, Yichun Zhou

**Affiliations:** ^1^ School of Advanced Materials and Nanotechnology, Xidian University, Xi’an 710071, China.; ^2^ Beijing National Laboratory for Condensed Matter Physics, Institute of Physics, Chinese Academy of Sciences, Beijing 100190, China.; ^3^ School of Materials Science and Engineering, Xiangtan University, Xiangtan 411105, China.; ^4^ School of Materials Science and Engineering, Tsinghua University, Beijing 100084, China.

## Abstract

HfO_2_-based ferroelectrics have evoked considerable interest owing to the complementary metal–oxide semiconductor compatibility and robust ferroelectricity down to a few unit cells. However, the unique wake-up effect of HfO_2_-based ferroelectric films severely restricts the improvement of their performance. In particular, the domain structure is an important characteristic of ferroelectric materials, which still has not been well understood in HfO_2_-based ferroelectrics. In this work, a Hf_0.5_Zr_0.5_O_2_ ferroelectric thin film is grown on a typical Si substrate buffered with TiN electrode. The 90° domains of the *Pca*2_1_ ferroelectric phase with head-to-tail and tail-to-tail structures can be observed by *C*s-corrected scanning transmission electron microscope under their pristine condition. After waking up, the 180° domain is displayed in the ferroelectric phase. The remarkable differences in domain walls for 90° and 180° domains are characterized by qualitatively mapping the polarization distributions at the atomic scale. The domain wall changes from the $\left[10\bar{1}\right]$ of the Hf_0.5_Zr_0.5_O_2_ film to the [001] of the Hf_0.5_Zr_0.5_O_2_ film. This result provides fundamental information for understanding the domain structure of HfO_2_-based ferroelectrics.

## Introduction

The experimental discovery of ferroelectricity more than a hundred years ago [1,2] has attracted great attention across many fields, such as their application in nonvolatile memories, infrared detectors, and tunable microwave devices [3–5]. Unlike traditional perovskite ferroelectric materials for which the ferroelectric properties deteriorate greatly when reduced to the unit cell scale (due to the depolarizing electric field caused by the interfaces [6–8]), HfO_2_-based films have been shown to exhibit robust ferroelectricity [9] and switchable polarization with film thicknesses as low as 1 nm [10,11]. In addition, HfO_2_-based ferroelectric thin films are compatible with complementary metal–oxide semiconductor technology and thus have great potential for device miniaturization [12,13]. Despite the huge potential of HfO_2_-based ferroelectrics [14–16], the unavoidable wake-up effect (namely, an increase in switchable polarization during the first 10^4^ to 10^5^ cycles) seriously restricts the uniformity and stability of the storage window in HfO_2_-based ferroelectric memory [17,18]. The exact mechanism of wake-up is still debated lamentedly. At present, the method for improving the wake-up effect in HfO_2_-based ferroelectric films focuses on doping, top electrode restriction, and rapid annealing [19]. Several mechanisms have been suggested previously to explain the origin of the wake-up effect. For example, Chouprik et al. [20] attributed the wake-up effect to the phase transition from the monoclinic phase to the orthorhombic phase based on the results of piezoresponse force microscopy (PFM) and transmission electron microscopy (TEM). Cheng et al. [21] believed that the films were transformed completely from the antipolar phase (*Pbca*) to the orthorhombic phase (*Pbc*2_1_) after waking up. Hence, it is generally accepted that the HfO_2_-based film becomes a ferroelectric orthorhombic phase after waking up, which is the metastable noncentrosymmetric orthorhombic phase in HfO_2_. More deeply, there are multiple polarization states in the metastable noncentrosymmetric orthorhombic phase, and these different polarization directions form different domain structures, such as the 90° and 180° domains [22]. Chouprik et al. [23] studied the domain structure of Hf_0.5_Zr_0.5_O_2_ (HZO) thin films in the wake-up effect process using PFM. It was found that an abnormal domain turns into a static domain and a normal domain becomes orderly, and the number of reversible domains tends to increase with the increases in the external electric field in the cycling process. Zhou et al. [24] suggested that the wake-up effect originates from the depinning of domains due to the reduction of the defect concentration near the TiN electrode. Therefore, investigating the variation of domains and domain walls (DWs) during the process of waking up is critical to understand the impact of microstructure on the wake-up effect at the atomic scale. In addition, a deep understanding of the atomic scale domain microstructure is of great importance. However, according to the above-mentioned work in the literature, it is difficult to study the atomic scale microstructure of domains by means of PFM, x-ray diffraction (XRD), etc.

In recent years, scanning transmission electron microscope (STEM) has become an effective way to characterize the DWs in HfO_2_-based thin films. On the basis of the above-mentioned analysis, the DW transitions of HfO_2_-based thin films may be a reason for the wake-up effect under bias voltage conditions. This phenomenon, however, has scarcely been reported in the literature. For instance, Grimley et al. [25] found the presence of 90° DWs in orthorhombic HfO_2_ grains and characterized misfits between different orthorhombic/orthorhombic domains. Several types of 90° and 180° DWs have also been observed in Y-doped HfO_2_ ferroelectric thin films grown on Y-stabilized ZrO_2_(100) substrates by Kiguchi et al. [26]. Xu et al. [27] has showed rich 90°/180° ferroelectric domains in massive single-crystalline HfO_2_:Y. Shimizu et al. [28] found the transformation of 90° DWs along the *b*-axis out-of-plane direction to the *c* axis when using an electric field. According to the analysis, the abovementioned works of the DW structure only detect the presence of Hf atoms using high-angle annular dark-field imaging (HAADF)-STEM, while the microstructure of domains includes O atoms in HfO_2_ [11]. It is therefore necessary to observe O atoms to further study the microstructure of domains.

In this study, we directly visualize the displacement of O atoms for orthorhombic *Pca*2_1_ in HZO ferroelectric thin films by integrated differential phase contrast (iDPC) STEM technology, which enables reliable imaging of light atomic columns (i.e., oxygen) in the presence of heavier atomic columns (i.e., Hf and Zr) in fluorite-structured HZO films. The 90° DWs with no charge and negative charge can be found at the atomic scale in pristine HZO films. The distinction can be qualitatively plotted by the polarization distributions across the 90° DWs, demonstrating the uncharged DW (UCDW) and negatively charged DWs (NCDWs) along $\left[10\bar{1}\right]$of the HZO film. After waking up, the pinched hysteresis curve transitions to a normal shape and the ferroelectric remnant polarization (2Pr) value increases from 12 to 28 μC/cm^2^ after waking up. Furthermore, the domain-wall structural evolution has occurred, which forms the 180° DWs along [001] of the HZO film.

## Results

**Fig. 1.**
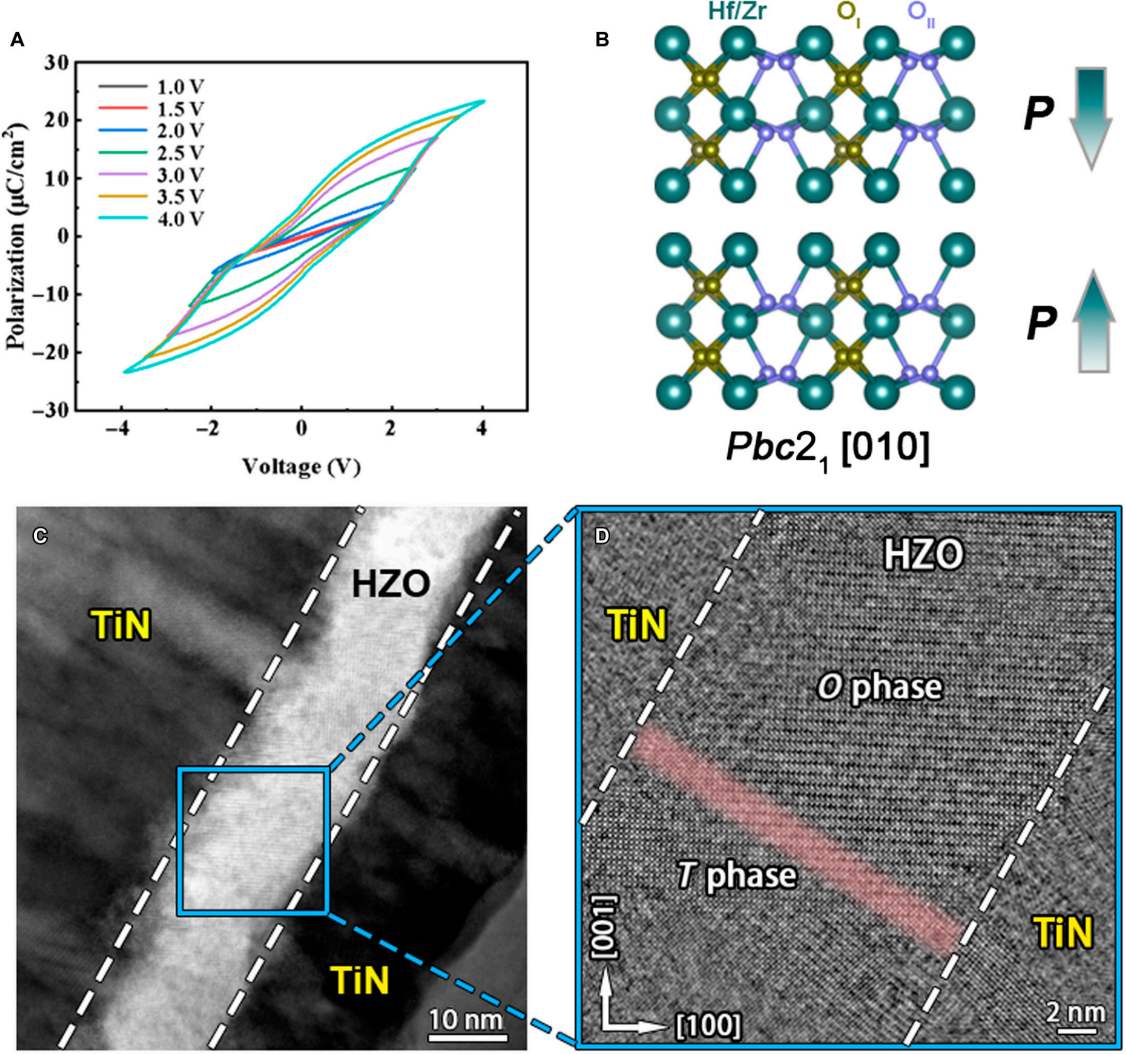
(A) Typical *P*–*V* loops of the HZO thin film. (B) The schematic unit cell of ferroelectric (Hf/Zr)O_2_. The atrovirens, yellow- and purple-colored solid balls, denote Hf/Zr, O_I_, and O_II_ atom columns, respectively. (C) Cross-sectional STEM image of the TiN/HZO/TiN stack. (D) Low-magnification iDPC-STEM image of the HZO ferroelectric thin film projected along *O*_[010]_ zone axes.

Figure 1A shows the polarization–voltage (*P*–*V*) loops of the TiN/HZO/TiN capacitor measured at 1 kHz under different voltages, which exhibits pinched hysteresis behavior. The 2Pr of 12 μC/cm^2^ is obtained at 4 V, demonstrating the ferroelectricity of the prepared HZO thin films. Figure 1B is a schematic of the ferroelectric phase HZO unit cell along [010] zone axis, in which 2 kinds of oxygen atoms (O_I_ and O_II_) with different displacement behaviors are denoted [11]. The O_I_ is the centrosymmetric oxygen atom, while O_II_ atoms are shifted as *δ*_O_ along *c* axis relative to the surrounding Hf tetrahedron center. As a consequence of this structure in which the negative (O_II_) and positive (Hr or Zr) charges are separated, a charged dipole is formed in the HZO unit cell. The polarization direction is in the opposite direction to the acentric oxygen atomic displacements (*δ*_O_), which points from the negative charge to the positive charge. For the HZO lattice, only one direction can maximize the difference to identify the different orthorhombic phases (*O* phase) because of their structural feature. Hence, [010] is identified as the most suitable zone axis to distinguish monoclinic, orthorhombic, and tetragonal phases. It is also relatively easy to identify the differences in the oxygen atom arrangements. The *O* phase also exhibited different polarization orientations, namely, the ferroelectric DWs may be formed. Figure 1C shows the HAADF-STEM image of the TiN/HZO/TiN stack, in which the interface indicated by a dashed white box between the HZO thin film and TiN metal electrodes is visible. The polycrystalline nature of the HZO thin film can be found and also observed from the collected XRD results (Fig. S5). Figure 1D provides an enlarged view of the blue frame indicated in Fig. 1C. A previous study reported the presence of periodic short–long Hf–Hf bonding along the *a* axis in the *O* phase [29]. Here, the *O* phase and the tetragonal phase (*T* phase) can be identified in certain directions based on the apparent lattice parameter differences between the corresponding lattice planes. As shown in Fig. 1D, orthorhombic [010] (the top part) and tetragonal [010] (the bottom part) are clearly identified, indicating the coexistence of these phases and the multiphase nature of the HZO film.

**Fig. 2.**
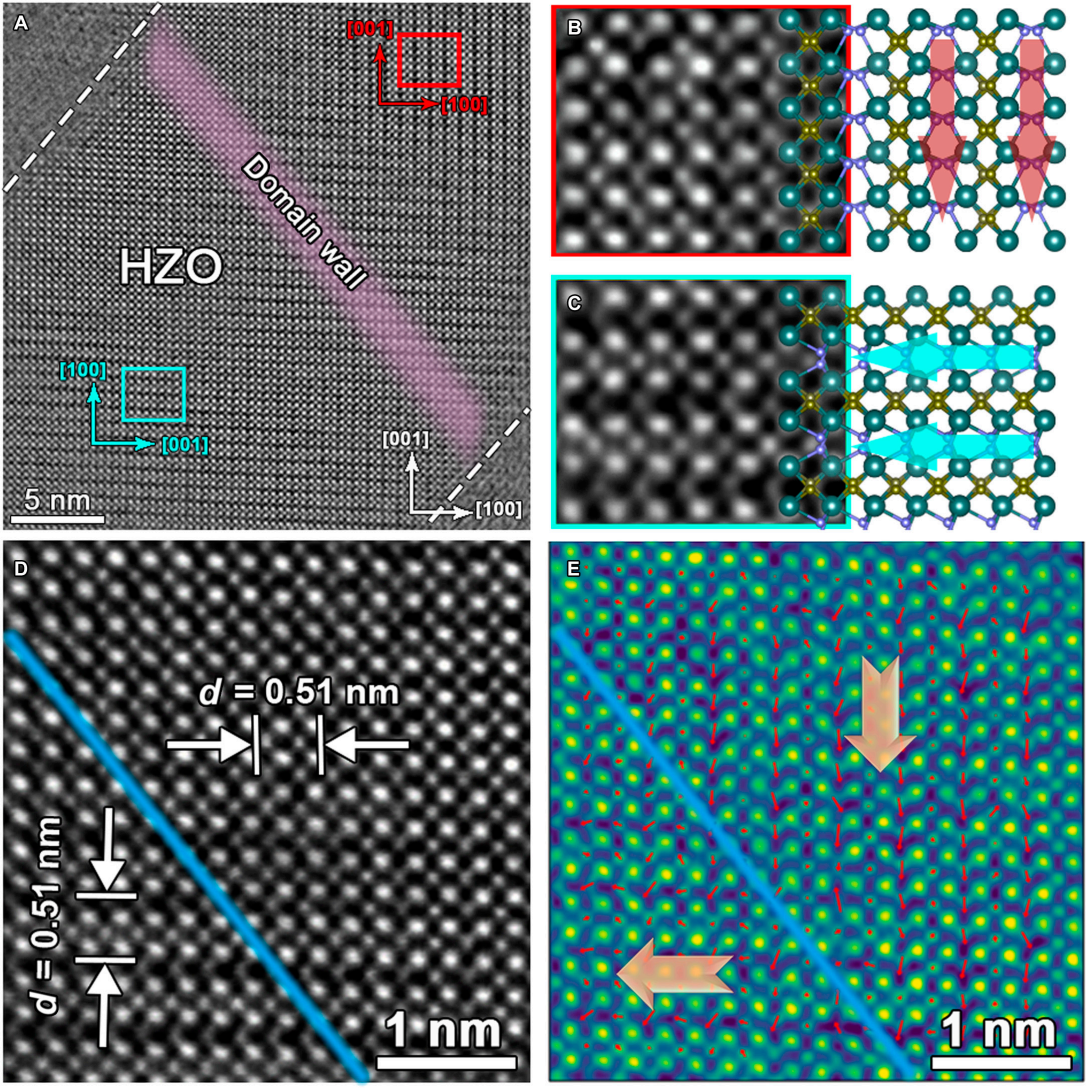
(A) High-magnification iDPC-STEM image of the HZO film, where the dashed white lines indicate the interfaces between the HZO and TiN electrodes. (B and C) Enlarged drawings obtained from the selected red and green areas highlighted in (A), where the arrows indicate the corresponding direction of polarization. (D) An atomically resolved iDPC-STEM image of the area obtained from (A). (E) Mappings of the *P*s vectors across the 90° UCDW that shows "head-to-tail" arrangement.

To elucidate the ferroelectric nature in the orthorhombic phase, hafnium/zirconium (Hf/Zr) and oxygen (O) atoms were directly detected using the atomic-scale iDPC technique that is sensitive to light elements such as oxygen [30]. The atomically resolved positions of Hf/Zr and O atoms are determined using iDPC-STEM, as shown in Fig. 2A. The strong contrast is associated with the heavy Hf/Zr atoms and light O atoms, projecting that the zone axis along the [010] is suitable. For the *O* phase, the displacement of O_II_ can be distinguished clearly. To further verify the shift of O_II_, the parts of the different regions (red and green frames) were amplified and studied, as shown in Fig. 2B and C. According to the high-magnification images, the periodic long–short of Hf–Hf bonding along the *a* axis for the *O*-phase unit cell also rotates 90° between the red and green frames. It includes the overlaid model using the *O* phase with *Pca*2_1_ space group. Since the polarization direction is opposite to *δ*_O_, the polarization direction is downward as shown in Fig. 2B and leftward as shown in Fig. 2C, which are shown by broad arrows in the model. Fast Fourier transform (FFT) operations (Fig. S1A and B) of the red and green selected areas also indicate a 90° rotation. On the basis of the orientation and size of the *δ*_O_ shift, the DWs can be confirmed with the light purple belt in Fig. 2A. To elaborate on the 90° DW in detail, the local area across the DW is magnified and shown in Fig. 2D with the location of the DW indicated by the blue line. Because of the differences between O_I_ and O_II_, the lattice constant appears as periodic black and white stripe columns along the *O*_[100]_ as shown in Fig. 2A, which fits well with the asymmetric *Pca*2_1_ structure. The lattice parameter mappings in Fig. S1C and D are in accordance with the characteristics of the Hf–Hf bonding. To study the microstructure in detail, Fig. 2E shows the polarization mapping corresponding to Fig. 2D, which was obtained by fitting the image with 2-dimensional Gaussian functions [31]. No extra substrate information is used to rectify the results, so qualitative analysis and discussion are carried out in the present work. The arrows located at the O column positions indicate the directions and distance of the spontaneous polarization (*Ps*) vectors (opposite to the *δ*_O_ vectors). Here, the O_II_ columns deviate from the central position of the 4 nearest Hf/Zr atoms, while the O_I_ columns shift only slightly. As emphasized in the schematic map of the HfO_2_ unit cell from Fig. 1B, the 3-fold shift of O_II_ is the origin of ferroelectricity, while the 4-fold O_I_ represents paraelectric behavior. The 90° DW can be observed clearly, and the directions of the *P*s vectors are sharp and possess an abrupt change in the projected symmetry at the DW.

**Fig. 3.**
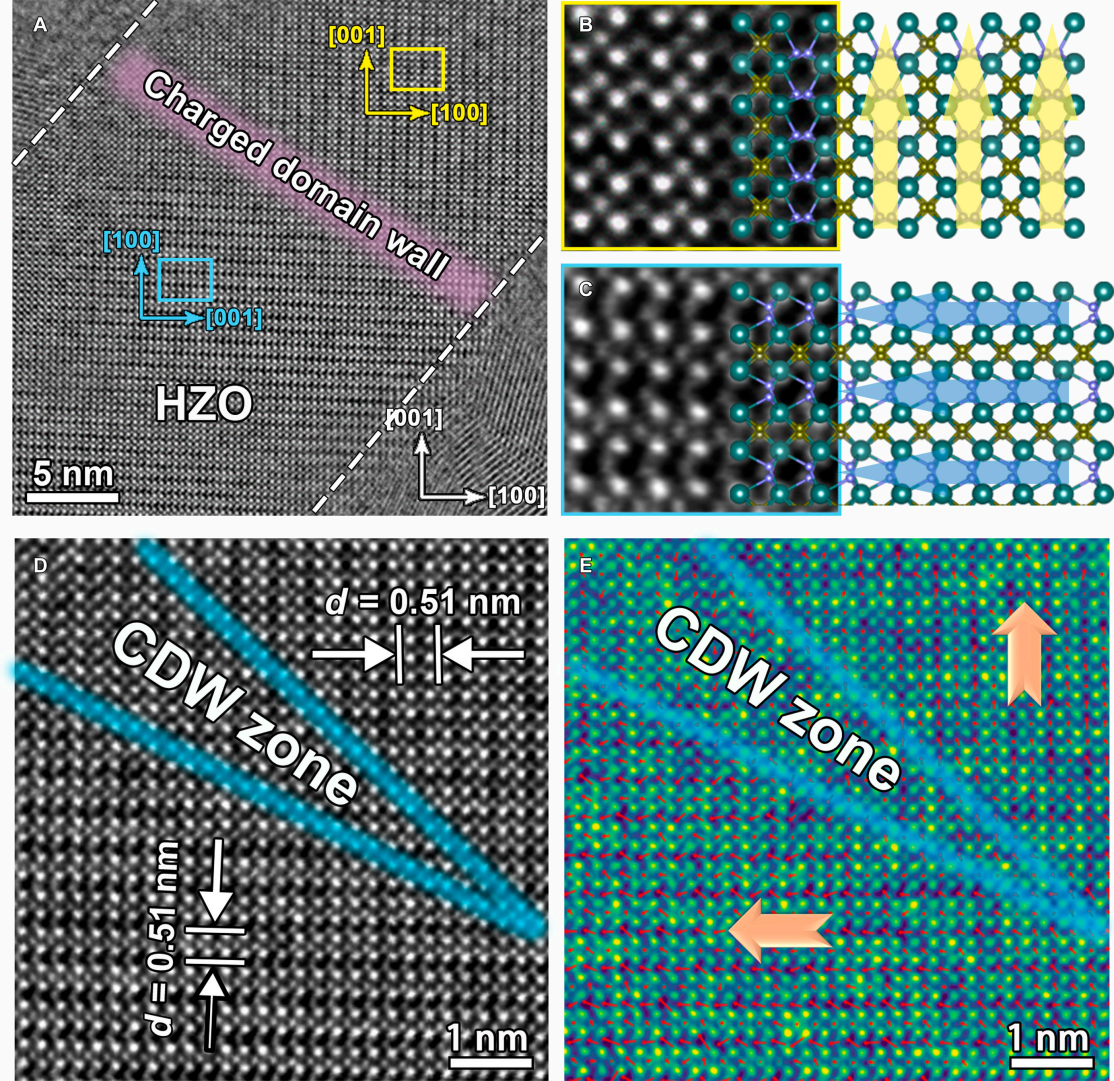
(A) High-magnification iDPC-STEM image of the HZO thin film, where dashed white lines indicate the interfaces between the HZO and TiN electrode. (B and C) Enlarged drawings obtained from the selected yellow and blue areas in (A). (D) An atomically resolved iDPC-STEM image of the area obtained from (A). (E) Mappings of the *Ps* vectors across the 90° NCDW that shows "tail-to-tail" arrangement.

Figure 3A shows an atomically resolved iDPC-STEM image of the HZO thin film containing a different DW structure. Similar to the methodology as shown in Fig. 2A, the 90° DW also exists. Figure 3B and C shows the enlarged images of yellow and blue frames, respectively, highlighted in Fig. 3A. The *δ*_O_ is shifted downward in Fig. 3B and rightward in Fig. 3C, generating the 90° DW with the tail-to-tail arrangement. In ferroelectric materials, in addition to the 90° DW “head-to-tail” arrangement, there are also 90° *P*s vectors with a “tail-to-tail” configuration. These “tail-to-tail” 90° DWs may induce negative bound charges [32], which are herein referred to as 90° NCDW. The DW of “head-to-tail” configuration are herein abbreviated as 90° UCDW. In the diffractograms by fast FFT (Fig. S2A and B), the *c* axis also rotates 90° between 2 images and that in Fig. 2. Hence, there is a 90° NCDW, which is outlined by the light purple belt in Fig. 3A. To clarify the DW structure, the area nearby to NCDW is magnified and displayed in Fig. 3D. According to the displacement of O_II_, NCDW is roughly depicted by the blue line shown in Fig. 3D. In-plane and out-of-plane lattice mappings (Fig. S2C and D) of Fig. 3D were also analyzed, which further confirms the similar structure characteristics in the 90° DW. To gain insight into the polarization distribution directly, the *P*s vectors of each unit cell near the 90° NCDW are also mapped and superimposed in Fig. 3E, where the 90° tail-to-tail polarization distributions can be observed. The directions of the *P*s vectors have changed rapidly in the 90° UCDW according to Fig. 2. By contrast, the changes of polarization is surprisingly slow across the 90° NCDW, resulting in no sudden jump in polarization direction. Hence, the 90° NCDW here is a broad area where the *P*s vectors are disordered and no apparent DW exists.

As shown in Fig. 2, the polarization direction of the domains is along $\left[\bar{1}00\right]$ and $\left[00\bar{1}\right]$ orientations in the HZO film, resulting in the UCDW observed along the $\left[10\bar{1}\right]$ of the HZO film. The location of the 90° NCDW is still along the $\left[10\bar{1}\right]$in the HZO film, which is consistent with the out of plane of the TiN/HZO/TiN capacitor, and also in the same direction as the thin film growth direction. Figure S3 schematically shows the distribution of polarization and the direction of the 90° NCDW and UCDW. In the UCDW, the polarization changes rapidly across the DW in Fig. 2, showing the same twinning characteristics as in tetragonal ferroelectrics [33]. Different from tetragonal ferroelectric materials, the 90° UCDW has an alternating spacer layer and ferroelectric layer [11] in HfO_2_, and the polarized charges are also produced at intervals. Thus, the alternating spacer layer and ferroelectric layer are strictly symmetric on either side of the DW, as displayed in Fig. 2. The charges tend to accumulate at the DWs when the above symmetry is broken, which may be one of the reasons for forming the CDW. The *Ps* is strongly restricted and disordered in Fig. 3, showing some unusual characteristics. Another reason for these observations is the bound charges produced at the 90° NCDW. It has been reported that internal charge carriers, such as electron holes or oxygen vacancies, can not only screen the bound charges emerging at the CDW [34] but also stabilize the CDW. In such HZO films, the charge carriers may be oxygen vacancies, which have a positive charge and are easily generated during growth [35]. Negative charge accumulation generated by the NCDW stabilizes the aggregation of oxygen vacancies and thus disturbs the 90° NCDW. This inference is consistent with our experimental observation. Recently, Petralanda et al. [36] have reported that oxygen vacancies promote the formation of the CDW in BaTiO_3_ by ab initio, which may support our conclusion.

**Fig. 4.**
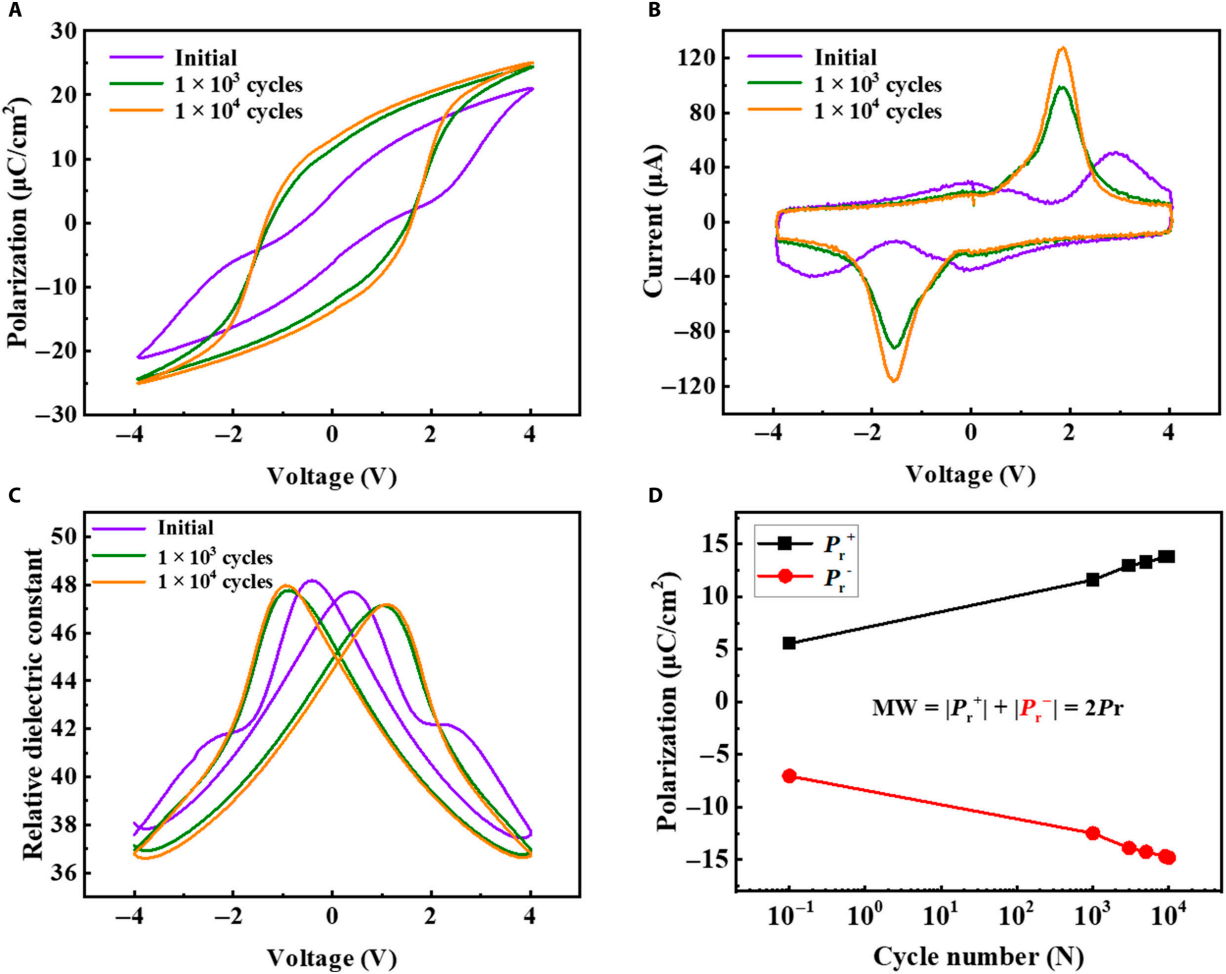
(A) *P*–*V* curves and (B) currents versus voltage (*I*–*V*) curves under different cycle numbers. (C) Relative dielectric constant (*ε_r_*) versus voltage curves, before and after waking up. (D) Pr values of the HZO film under different cycle numbers. MW, memory window.

As mentioned above, the wake-up effect influences the uniformity of storage windows, which is under continuous switching cycles. Figure 4A to C shows the *P*–*V* curves, currents versus voltage (*I*–*V*), and relative dielectric constant (*ε_r_*) versus voltage (*ε_r_*–*V*) curves obtained under the same working conditions as indicated in the figures. As shown in Fig. 4A, the polarization–voltage curve gradually opens along with the enhanced polarization values with the increase in the electric field cycles. The double hysteresis curve becomes a typical shape when the electric field cycles reach up to 1 × 10^4^ times, showing a *P*–*V* curve with good rectangularity. In Fig. 4B, it can be found that the variation trend of current is consistent with polarization (Fig. 4A) and the polarization reversal current is also in accordance with the *P*–*V* curve. Figure 4C shows the *ε_r_*–*V* graph of the HZO film before and after waking up, which has the typical butterfly shape. The peak of *ε_r_* on the *ε_r_*–*V* curves corresponds to the coercive field on the *P*–*V* curve, which further proves that the HZO film has excellent ferroelectricity. The larger the closed area formed by the *ε_r_*–*V* curve, the greater the contribution of the ferroelectric domain inversion under this condition, and the stronger the ferroelectricity. It can also be seen that the *ε_r_*–*V* curves of the HZO film show a typical butterfly shape after waking up. Figure 4D shows the variation of 2Pr for a TiN/HZO/TiN capacitor with increasing numbers of switching cycles, which shows the presence of a distinct wake-up effect. The 2Pr value increases from 12 to 28 μC/cm^2^ after waking up. From PFM (Fig. S4), it is also verified that the ferroelectricity is improved.

At present, according to the preface, there are 2 main explanations for the wake-up effect. One is due to the field-induced phase transition in the interface region and the interior of the film after being activated by the electric field. Specifically, the tetragonal phase is transformed into the orthogonal ferroelectric phase *Pca*2_1_, and the macroscopic manifestation is an increase in the remanent polarization value. Another explanation is the diffusion and redistribution of oxygen vacancies and defects in the interface region under the activation of the electric field, which weakens the built-in bias, resulting in the depinning of the domains and the increase in the remanent polarization value. The initial *P*–*V* curve possesses a pinched hysteresis, which disappears with the increasing cycles. Before waking up, there are 90° UCDWs and 90° NCDWs, which is the reason for the appearance of a pinched hysteresis [37]. Therefore, TEM can be used to study the domain induction mechanism for the changes observed in Fig. 4 after waking up.

**Fig. 5.**
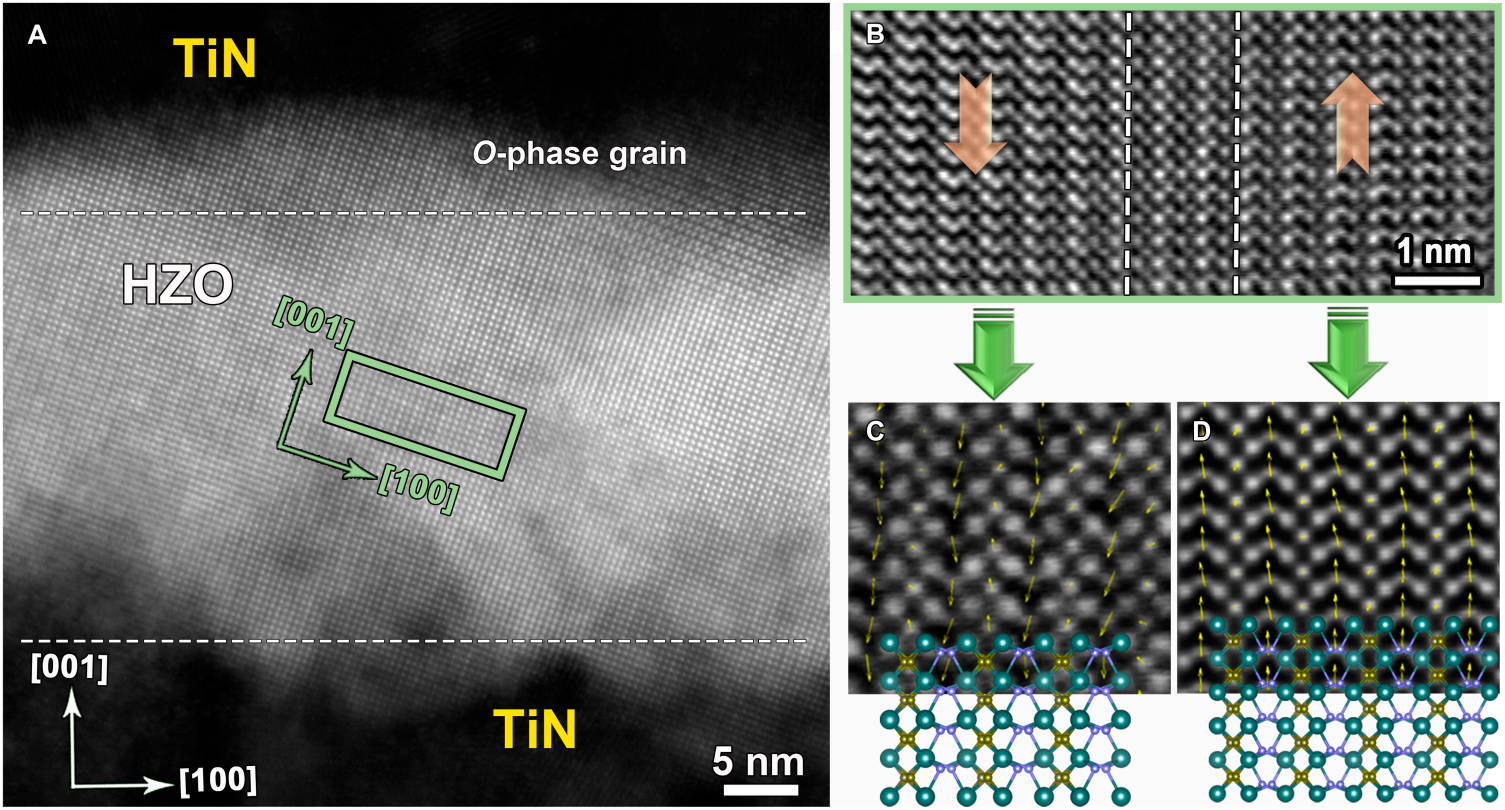
(A) An atomically resolved HAADF-STEM image of the TiN/HZO/TiN stack after waking up. (B) iDPC-STEM image acquired from the green square area in (A), the O_II_ atomic columns of the left image shifted along the [001] direction, and the O_II_ atomic columns of the right image shifted along the $\left[00\bar{1}\right]$ direction. The polarization mapping of the left image (C) and the right image (D) in (B).

The HAADF-STEM image of the sample after waking up is shown in Fig. 5A. Here, an *O* phase is projected along the [010] zone axes of the HZO film. It is clear that the periodic long–short Hf–Hf bonding is along the *a* axis. Figure 5B shows the corresponding iDPC-STEM image of the green frame in Fig. 5A. The location of oxygen atoms can be observed clearly. The O_II_ atomic columns on the left shift along the [001] direction, while the opposite O_II_ atomic columns on the right shift along the $\left[00\bar{1}\right]$ direction. Thus, there is 180° DW as is marked by a dashed white line in Fig. 5B. The 180° DW contains 2-unit cells unexpectedly, which is different from the 90° UCDW and 90° NCDW. A 2-dimensional fitting is also used to obtain the polarization information, as shown in Fig. 5C. The conversely polarized arrows of the fitting results further confirm the presence of a 180° DW. In addition, the 180° DW of the HZO film in Fig. 5 is perpendicular to the (001) plane of the HZO, which is close to the out-of-plane direction of the TiN/HZO/TiN film. Therefore, the wake-up effect could be relevant to the DW transition from the 90° DW in the pristine sample to the 180° DW after electric field cycling. This is a reasonable interpretation of the DW transition in the wake-up effect due to the fact that the polarization is determined by the domain structure for ferroelectrics. As mentioned earlier, oxygen vacancies are present in HZO films before waking up, which can then be redistributed under the action of electric field cycling. The redistribution of the oxygen vacancies is also one reason for the evolution of the domain structure. In addition, the 180° DW has negative DW energy and sufficient migration barrier [38], so the 90° domain is easy to translate to the 180° domain under the action of an electric field.

## Discussion

In conclusion, *C*s-corrected iDPC-STEM is exploited to analyze the dipole arrangement behavior of the 90° DW and CDW before waking up and the 180° DW after waking up in an orthorhombic HZO thin film at the atomic scale. The polarization distributions across these DWs are then plotted quantitatively. Crystallographic analysis indicates that the 90° DWs are all along the $\left[10\bar{1}\right]$ of the HZO flm. The evolution of domain structure can be observed with the increase in electric field cycling. After waking up, the 180° DW along the [001] of the HZO film appeared in this study. According to the results, it can be found that the wake-up effect will occur when the DW type and DW plane have changed in the HZO film. The disappearance of a pinched hysteresis is accompanied by a variation of 90° DW to 180° DW. With regard to the 90° NCDW, the appearance of unordered dipole behaviors can be attributed to the stabilization of oxygen vacancies in HZO ferroelectric thin films. These results visualize the domain configuration evolution in HZO ferroelectric thin films, which helps to further investigate the intrinsic nature of the ferroelectric *O* phase and the polarization evolution mechanism.

## Materials and Methods

The 15-nm-thick HZO thin films were grown on TiN electrodes using a HfO_2_ and ZrO_2_ cycle ratio of 1:1 by atomic layer deposition at 260 °C. Here, the TiN electrode was deposited onto the cleaned and heavily doped P-type silicon wafer. Tetrakis-dimethylamido-hafnium {Hf[N(CH_3_)_2_]_4_}, tetrakis-dimethylamido-zirconium {Zr[N(CH_3_)_2_]_4_}, and H_2_O precursors were used as the Hf and Zr precursors and oxidant, respectively. After the deposition of HZO thin films, TiN top electrodes were formed with a stencil mask by magnetron sputtering. Last, crystallization annealing was performed at 450 °C for 30 s in an N_2_ atmosphere by rapid thermal annealing. The ferroelectric properties of the HZO thin films were measured by aixACCT TF Analyzer 3000. The times of the circulating electric field are 1 × 10^3^ and 1 × 10^4^, respectively. Cross-sectional TEM samples were prepared by the focused ion beam technique. The pristine TEM sample was obtained from the unawakened top electrodes, and the wake-up TEM sample was obtained from the awakened top electrodes. The iDPC-STEM images were acquired using a Titan Cubed 60- to 300-kV microscope (FEI).

## Data Availability

The data that support the findings of this study are available from the corresponding author upon reasonable request.

## Supplementary Materials

Supplementary 1 Fig. S1. (A and B) FFT diffractograms obtained from Fig. 2B and C. Fig. S2. (A and B) FFT diffractograms obtained from Fig. 3B and C. Fig. S3. (A) and (B) are the polarization distribution sketch maps of the 90° UCDW and 90° NCDW. Fig. S4. (A and B) PFM phase (A) and local PFM hysteresis loops (B) of a pristine capacitor; (C and D) PFM phase (C) and local PFM hysteresis loops (D) after the waking-up process. Fig. S5. The XRD of the 2 TiN/HZO/TiN samples which are a pristine capacitor (2) and after the waking-up process (1).
