# Supplementary material for: Domain Wall Evolution in Hf0.5Zr0.5O2 Ferroelectrics under Field-Cycling Behavior
Source: Research (Wash D C). 2023 Mar 28;6:0093. doi: 10.34133/research.0093 (PMC11789571; doi:10.34133/research.0093)
Supplement: Supplementary 1 — Fig. S1. (A and B) FFT diffractograms obtained from Fig. 2B and C. Fig. S2. (A and B) FFT diffractograms obtained from Fig. 3B and C. Fig. S3. (A) and (B) are the polarization distribution sketch maps of the 90° UCDW and 90° NCDW. Fig. S4. (A and B) PFM phase (A) and local PFM hysteresis loops (B) of a pristine capacitor; (C and D) PFM phase (C) and local PFM hysteresis loops (D) after the waking-up process. Fig. S5. The XRD of the 2 TiN/HZO/TiN samples which are a pristine capacitor (2) and after the waking-up process (1). [file research.0093.f1.docx]

**Title**

Domain Walls Evolution in Hf_0.5_Zr_0.5_O_2_ Ferroelectrics under Field-Cycling Behavior

**Authors**

Sirui Zhang^1^, Qinghua Zhang^2*^, Fanqi Meng^2^, Ting Lin^2^, Binjian Zeng^3^, Lin Gu^4^, Min Liao^1*^ and Yichun Zhou^1*^

**Affiliations**

^1^School of Advanced Materials and Nanotechnology, Xidian University, Xi’an 710071, China

^2^Beijing National Laboratory for Condensed Matter Physics, Institute of Physics, Chinese Academy of Sciences, Beijing 100190, China

^3^School of Materials Science and Engineering, Xiangtan University, Xiangtan 411105, China.

^4^School of Materials Science and Engineering, Tsinghua University, Beijing 100084,China.

Correspondence should be addressed to Qinghua Zhang; [zqh@iphy.ac.cn](mailto:zqh@iphy.ac.cn) Min Liao; [mliao@xidian.edu.cn](mailto:mliao@xidian.edu.cn) and Yichun Zhou; yichunzhou@xidian.edu.cn


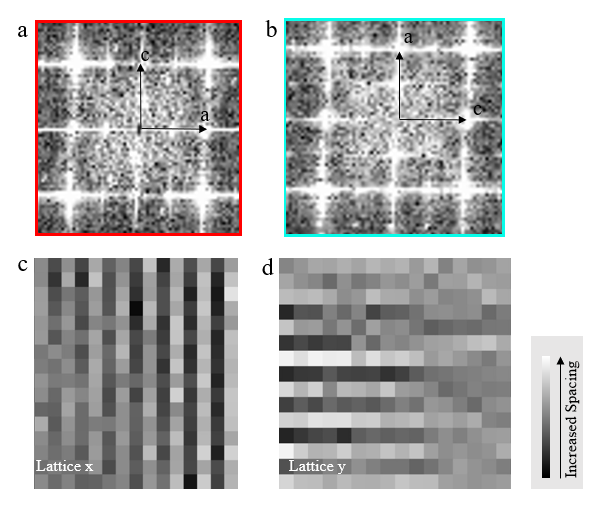


**Fig. S1.** a)-b) The FFT diffractograms obtained from the Figure 2b and 2c. The lattice x c) and the lattice y d) mappings of Figure 2d. In in-plane lattice map of Figure S1c, periodic stripe can be found in the upper right along horizontal direction, so the [001] orientation of this region is along vertical direction that is out-of-plane orientation. In out-of-plane map of Figure S1d, periodic stripe can be found in the lower left as well as that in Figure S1c, but this stripe is along vertical direction, indicating the [001] orientation of this region is along horizontal direction. Then the 90° domain is further revealed in lattice parameters.


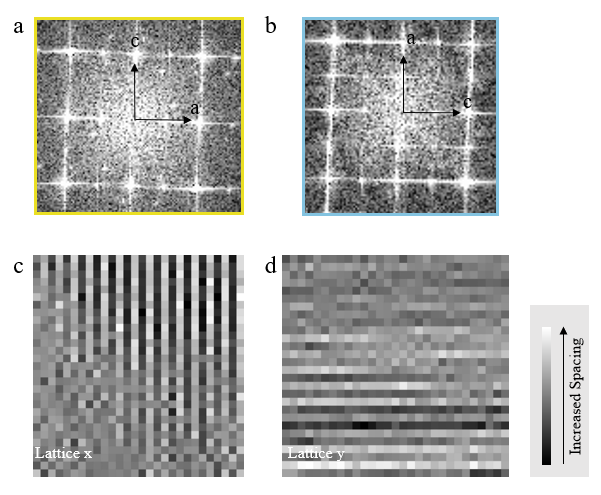


**Fig. S2.** a)-b) The FFT diffractograms obtained from the Figure 3b and 3c. The lattice x c) and the lattice y d) mappings of Figure 3d. In-plane and out-of-plane lattice mappings of Figure 3d are analyzed in Figure S2c and S2d. Further confirms that structure characteristic in this 90° domain wall.


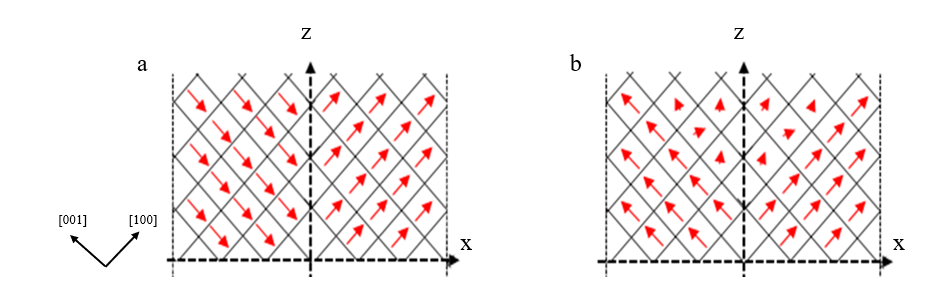


**Fig. S3.** a) and b) are the polarization distribution sketch map of the 90° UCDW and 90° NCDW. The z direction represents the growth direction of the HZO thin film, which is consistent with the <110> direction of the HZO film.


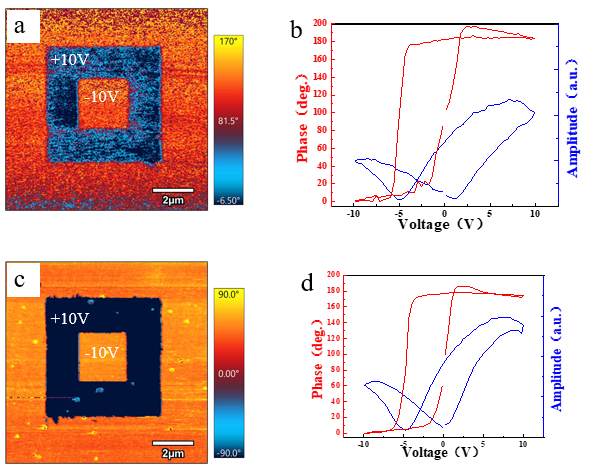


**Fig. S4.** a)-b) PFM phase a) and local PFM hysteresis loops b) of a pristine capacitor; c)-d) PFM phase c) and local PFM hysteresis loops d) after the waking up process.


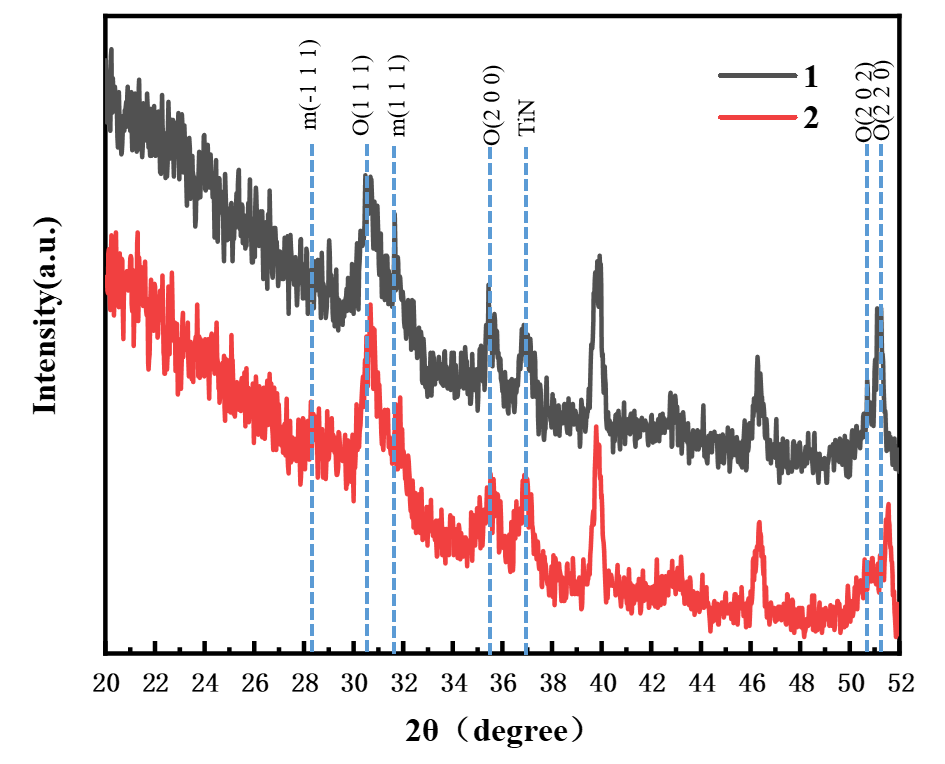


**Fig. S5.** The XRD of the two TiN/HZO/TiN samples which are a pristine capacitor (2) and after the wake-up process (1).
